# The Effect of Mobile Health Intervention on Prelacteal Feeding Among Mothers in the First Month After Birth in South Ethiopia: A Cluster-Randomized Controlled Trial

**DOI:** 10.3390/nu18111795

**Published:** 2026-06-02

**Authors:** Girma Gilano, Andre Dekker, Rianne Fijten

**Affiliations:** 1 Department of Public Health Informatics, School of Public Health, College of Medicine and Health Sciences, Arba Minch University, Arba Minch P.O. Box 21, Ethiopia; 2 Department of Radiation Oncology (Maastro), GROW Research Institute for Oncology and Reproduction, Maastricht University Medical Centre+, 6229 ER Maastricht, The Netherlands; andre.dekker@maastro.nl (A.D.); rianne.fijten@maastro.nl (R.F.)

**Keywords:** prelacteal feeding, mHealth, trials, breastfeeding, SMS, Ethiopia

## Abstract

**Introduction:** Prelacteal feeding, the practice of giving newborns substances other than breast milk within the first few days of life, remains a common yet harmful practice in many low- and middle-income countries, including Ethiopia. No evidence in Ethiopia indicates that mHealth can help improve prelacteal feeding. This study aimed to evaluate the effect of mobile health (mHealth) intervention on reducing prelacteal feeding practices and improving antenatal care (ANC) and postnatal care (PNC) utilization among mothers in South Ethiopia. **Methods:** A cluster-randomized controlled trial (CRT) was conducted in rural areas of South Ethiopia. A total of 20 clusters were selected using simple random sampling for intervention (mHealth) and control groups, each containing 340 women. Mothers in the intervention group received automated weekly SMS messages and reminders on exclusive breastfeeding, prelacteal feeding risks, ANC, and PNC. Mothers were only selected if they could read, write, and use mobile phones. **Results:** The mHealth intervention significantly reduced prelacteal feeding practice (AOR = 0.19, 95% CI: 0.06–0.58); *p* < 0.05). Higher ANC visits related to decreased prelacteal feeding (AOR = 0.28, 95% CI: 0.21–0.39; *p* < 0.001). The log count of ANC visit increased by 0.14 among intervention groups (IRR = 1.15, 95% CI: 1.06–1.25; *p* < 0.001). The PNC time was delayed 2.05 days among controls (β = −2.05, 95% CI: −2.66–−1.42; *p* < 0.001). Maternal and partner education, postnatal time, and ANC visits influenced prelacteal feeding. **Conclusions:** This finding might suggest that mHealth can reduce prelacteal feeding practices and improve maternal healthcare behaviors such as ANC attendance and timely PNC. These findings highlight the potential of mobile health interventions in promoting healthy maternal and infant practices in rural settings, where healthcare access is limited. Further research is needed to explore the long-term impacts of such interventions on maternal and child health outcomes. Multi-level analysis reduced variability. However, an unexplained variance could be reduced by including more cluster-level variables.

## 1. Introduction

Maternal and child health remain global public health priorities, especially in low- and middle-income countries (LMICs), where maternal mortality rates and infant morbidity are still high. The critical periods surrounding childbirth, antenatal, postnatal, and early neonatal stages require comprehensive healthcare interventions to mitigate risks of complications for both mother and child [1,2,3]. Among the postnatal harmful practices, prelacteal feeding, the practice of feeding newborns substances other than breast milk in the first few days of life, remains a widespread problem in low- and middle-income countries, despite its well-documented negative impact on breastfeeding practices and infant health [1,4]. Prelacteal feeding undermines early and exclusive breastfeeding, which is vital for reducing neonatal infections and promoting infant nutrition [5].

Addressing factors influencing prelacteal feeding requires understanding social, cultural, and healthcare-related factors [5,6]. Similarly, inadequate utilization of antenatal care (ANC) and postnatal care (PNC) services can increase the risk of adverse maternal and infant outcomes, including prelacteal feeding [5,6,7,8]. ANC allows healthcare providers to deliver crucial preventive and curative services, including iron supplementation, nutritional counseling, and guidance on newborn care. Studies have consistently shown that frequent ANC visits are associated with better maternal health outcomes, including reduced risk of harmful practices, such as prelacteal feeding [1,9]. However, barriers to accessing ANC and PNC services, such as geographic location, educational level of the mother, and healthcare availability, continue to affect maternal and infant health, related to prelacteal feeding [10,11]. Mobile health (mHealth)-based messages sent to mothers during ANC and PNC could improve these barriers [12].

In low- and middle-income countries, only one in two newborns receives breast milk in the first hour of life [13,14]. In Africa, prelacteal feeding remains a major public health problem in rural and urban areas. It is common in Western, Northern, and Southern African regions [15,16,17]. In sub-Saharan and East African countries, the pooled prevalence of prelacteal feeding was 32% and 12%, respectively [5,9]. In Ethiopia, the pooled prevalence of prelacteal feeding practices was 25.29% [18]. Despite efforts during the last decades, prelacteal feeding remains a public problem in Ethiopia. The current study introduces mHealth to provide information and reduce these abnormal feeding practices.

High maternal and infant mortality rates in Ethiopia require effective interventions addressing factors influencing maternal and child health behaviors, including education, prelacteal feeding, occupation, religion, rural residence, ANC utilization, and sociocultural factors [19,20,21]. Proper healthcare utilization can prevent maternal and child deaths. Prelacteal feeding rates have not improved (adequately) over time in Ethiopia: 26% in 2016, 25% in 2018, 21% in South Ethiopia [7,18,22], and 17% in the Gamo zone [23]. The current study extends beyond physical obstacles and fills the information gap on child feeding practices using mHealth.

### Objectives

Previous studies have shown that mobile health applications can address challenges and discrepancies in maternal healthcare behavior [24,25,26]. However, to our knowledge, no study directly used mHealth to address prelacteal feeding. One study in North Ethiopia indirectly showed reduced prelacteal feeding among mHealth intervention mothers [27]. Therefore, this study aims to assess the impact of mHealth intervention on reducing prelacteal feeding practices and improving ANC and PNC utilization in a rural Ethiopian population. The findings from this study can contribute to the growing body of evidence needed to inform maternal and child health policies and interventions in Ethiopia and similar settings.

## 2. Methods and Materials

### 2.1. Study Design

This study was conducted as a cluster-randomized controlled trial (CRT) to assess the effect of a mobile health (mHealth) intervention on the prevalence of prelacteal feeding among mothers in South Ethiopia. The study comprises two equal groups: intervention and controls, assessed at baseline and the end of each objective. The study clusters were primary health facilities (HF), including primary hospitals, health posts, and health centers. As detailed in the protocol [28], the HF must be functional and in the Gamo or Gofa zones to be included in the study. We do not have specific criteria for HF, but we randomized 20 of the 102 available HF. Although the WHO recommends more samples [29] for HF assessment, we assumed 20% (20/102) is sufficient for this study, depending on the study type. The study recruited participants from these health centers from 1 March 2024 to 30 April 2024. Simple random sampling was applied to select eligible women using the ANC registers and family folders. Women who met the eligibility criteria were invited to sign a participation consent before collecting baseline data. The analysis was conducted from May 2024 to September 2024. The details of the trial procedures were published online [28] and in other articles; here, we only summarize the main parts. The report followed CONSORT guidelines for cluster-randomized trials, and ethical approval was obtained from the Arba Minch University Institutional Review Board (AMU IRB), Ref. No 1326/2022. The study involved all women who provided informed consent through signature or fingerprint. We allowed participants to withdraw at any time without affecting the quality of care.

### 2.2. Rationale for Using Cluster-Randomized Trial (cRCT)

This study employs a two-arm, parallel-group, cluster-randomized controlled trial (cluster-RCT). The unit of randomization is the kebele (the smallest administrative unit in Ethiopia), which corresponds to a health post catchment area served by a pair of Health Extension Workers (HEWs). Cluster randomization was selected to minimize contamination between intervention and control groups, as women residing in the same kebele share a common HEW, social networks, and community health resources, making individual randomization impractical and methodologically unsound.

### 2.3. Study Setting

The trial was conducted in the Gamo and Gofa zones of South Ethiopia [previously the South Nation Nationalities and Peoples’ Region (SNNPR)]. Gamo and Gofa zones are predominantly rural; their maternal and child health services are often limited. The area has concerns about prelacteal feeding and low utilization of antenatal and postnatal care services [30], making it a suitable setting for investigating the impact of the intervention. A total of 20 HFs and their catchment were included and selected based on the criteria for selection as published in the protocol.

### 2.4. Participants

All eligible women in the area were approached. Eligible participants for this outcome were women aged 18 and above who resided in the selected area, were under follow-up since ANC, could read and understand messages, and had access to a mobile phone for the intervention. The women at 16–28 weeks of gestational age (GA) were recruited over two months. Recruitment began at 16 weeks of GA and concluded at 24–28 weeks of GA. The GA at recruitment ranged from 16 to 28 weeks, reflecting a recruitment period of two months. Initially, the intervention participants were between 24 and 28 weeks of gestation. All women who met these eligibility criteria were included.

The study excluded mothers with severe health conditions or complications during childbirth that required hospitalization beyond 48 h, as this time is beyond the prelacteal period. Two people outside the research team from the same university [31], and randomized clusters and individuals. For this specific objective, excluding these participants could introduce post-randomization bias. However, we retain all participants who remained in the study until the data collection period.

### 2.5. Sample Size

Step 1: Individual sample size (assuming simple random sampling).

The required sample size per arm for an individually randomized trial was calculated using the two-sample proportion formula:

n = [(Z_(α/2) + Z_β)^2^ × (P_0_ (1 − P_0_) + P_1_ (1 − P_1_))] × [1 +(n − 1)ρ]/ (P_1_ − P_0_)^2^

where

Design effect = [1 + (n − 1) ρ] = 1.37;

Effect size = P_1_ − P_0_ = 0.12;

Type I error (α) = 0.05 (two-sided), therefore Z_(α/2) = 1.96;

Power (1 − β) = 0.80 (80%), therefore Z_β = 0.84 and ICC of 0.011.

Proportion in the control group (P_0_) = 0.709 (70.9%). This is based on a study conducted in South West Ethiopia in 2021, which reported the prevalence of childhood vaccination among children under 6 months. The expected proportion in the intervention group was (P_1_) = 0.826 (82.6%). This represents a 12-percentage-point absolute increase, corresponding to a relative increase of 37.5%. This effect size is consistent with mHealth intervention effects reported in Ethiopia, where mobile health reminders increased childhood vaccination by 20% [31], and with findings from the scoping reviews of mobile health interventions on vaccination coverage in LMICs [32].

Now, the total required sample size has become 680 mothers. We recruited 340 participants per arm.

### 2.6. Randomization and Allocation Procedures

Clusters were randomly assigned to either the intervention or control arm (1:1) using a computer-generated random allocation sequence prepared by two independent statisticians who were not involved in participant recruitment or intervention implementation. Before randomization, all eligible clusters were identified and enrolled in the trial. Gamo and Gofa zones have 102 health facilities used as clusters. Health facilities were numbered from 1 to 102, with each number and name placed on a piece of paper inside an opaque box. Randomization was conducted at the cluster level to minimize contamination between study groups.

To ensure balance between trial arms, restricted randomization was applied based on key cluster characteristics, including geographic location and population size. Each cluster is in a different district with the same number of hospitals and health centers are maintained in both arms. In both the intervention and control groups, cluster types were maintained equally without additional stratification. We used public health facilities as clusters; the primary hospital serves 100,000 people, the health centers serve 25,000 people, and the health posts serve 5000 people. The birth and pregnancy remain at 4% in the general population [33]. Following sequence generation, clusters were assigned to the respective study arms according to the predefined randomization list.

Allocation concealment was maintained by keeping the randomization sequence inaccessible to field data collectors and recruitment personnel until assignment was completed. Investigators responsible for participant enrolment were not involved in generating the allocation sequence. Due to the nature of the intervention, blinding of participants and implementers was not feasible; however, outcome assessment and data analysis were conducted using standardized procedures to minimize bias. Data collectors and care providers were blinded to the allocation of exact outcome variables under observation.

During individual randomization, ANC registration numbers were utilized at primary hospitals and health centers, whereas family numbers were used at health posts.

### 2.7. Intervention

**Figure 1:**
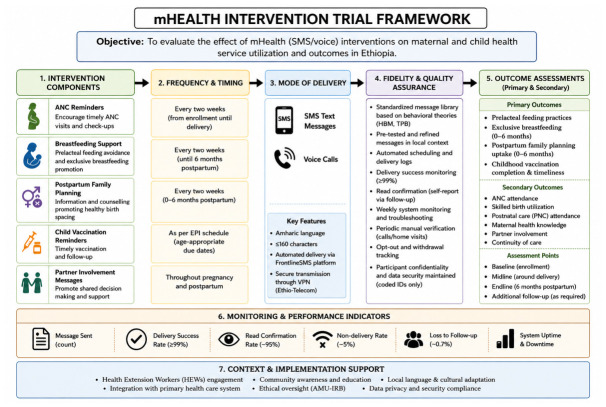
The diagram showing the intervention framework details.

The mobile health (mHealth) intervention sends weekly health information and appointment reminders to mothers in the intervention group for 5 months for this specific objective (Figure 1). The data was collected at the end of the first month post-delivery, with the other objective interventions continuing until the fifth month postnatal. The messages provided information on the benefits of EBF and complementary feeding, the risks associated with prelacteal feeding, and practical tips on newborn care, PNC, ANC, postpartum family planning, and childhood vaccination. The messages were developed from the World Health Organization guideline and adapted to the local context. Healthcare providers in selected HFs were unaware of the trial’s final participants and purpose. Overall, we planned to send 12,240 messages at the end of the project. For this specific objective, we sent 5408 over the intervention period. The fidelity or success rate is approximately 98.8%. The fidelity was not 100% due to some messages not being read or delivered, and unknown technical issues (‘not available’ back delivery after days of the message being sent). The main author creates the messages and tests them in the clusters not selected for the study. The trial manager is responsible for technical issues, software programming, and uploading messages to the already set schedule on the desktop computer installed in the health informatics department at AMU. The messages were initially prepared in English, for instance, “Giving a newborn baby anything other than a mother’s breast milk causes serious health problems,” and then translated into Amharic “አሁን ለተወለደ ህፅን ከእናት ጡት ውጪ ሌላ ነገር መስጠት ትልቅ የጤና ችግር ያመጣል”, since all intervention mothers speak and write Amharic. The messages were slightly limited to 160 characters. The messages were delivered at 7:00 a.m. local time and considered unsuccessful beyond 11:00 p.m.

### 2.8. Control Group

Mothers in the control group received standard care provided by local health services. This typically included a single postnatal visit and general breastfeeding advice without the targeted mHealth intervention.

### 2.9. Data Collection

Data were collected through face-to-face and phone interviews conducted by trained data collectors. Data collectors used a printed questionnaire for the phone and the ODK mobile app for face-to-face interviews. The primary outcome was the prevalence of prelacteal feeding within the first three days after childbirth, assessed one month postpartum. Secondary outcomes included the number of antenatal care (ANC) visits and time to postnatal care (PNC). Information on potential confounding variables, such as maternal education, occupation, and household income, was also collected. The data collection tool was adapted and validated from previous studies that used mHealth in Africa and Ethiopia [23,28,34,35].

### 2.10. Outcome Measures

Primary Outcome: For this objective, the primary outcome was the prevalence of prelacteal feeding, defined as any food or liquid (other than breast milk) given to the newborn within the first three days of life. The woman was directly asked “Given anything other than colostrum/breast milk during the first 3 days?” We applied multi-level logistic regression for this outcome. The women responded ‘yes’ if they were given any food or liquid and ‘no’ if they were not given anything within the first three days. Outcomes (objectives) such as childhood vaccination, postpartum family planning, and exclusive breastfeeding were reported elsewhere. The current study was based on one of the secondary outcomes of the doctoral project.

Secondary Outcomes:

ANC Frequency: The number of antenatal care visits attended during the most recent pregnancy. We used multi-level Poisson regression to account for this count data.

Time to PNC: The duration it took for the mother to attend her initial postnatal care visit. Multi-level linear regression was applied for this continuous variable.

### 2.11. Statistical Analysis

As stated above, we used multi-level logistic, Poisson, and linear regression models to account for the clustering of participants within health facilities (HFs). The multi-level analysis comprised four sequential models: (1) a null model (random-intercept only), containing only the outcome variable with no predictors; (2) a community-level model, including cluster-level variables; (3) an individual-level model (fixed-effects model), including only individual-level variables; and (4) the final model (mixed-effects model), which simultaneously included both cluster-level and individual-level variables. The effectiveness of the intervention was expressed as odds ratios (OR) with 95% confidence intervals (CIs) for prelacteal feeding, controlling for baseline differences between groups. Model fitness was assessed using the Akaike Information Criterion (AIC), Bayesian Information Criterion (BIC), and intra-cluster correlation (ICC). The analysis encompassed all participants who began and were established in the study, whether they were lost to follow-up or not. Further details are available in the protocol [28]. The study included mothers proficient in reading, writing, and manipulating their mobile phones. The mothers should manage their mobiles and messages themselves. The messages were sent through a VPN (Virtual Private Network) installed at the Arba Minch University Health Informatics Department. Per-protocol analysis was utilized, as four cases dropped had not commenced the intervention, despite the consortium’s intent-to-treat expression.

## 3. Results

**Figure 2:**
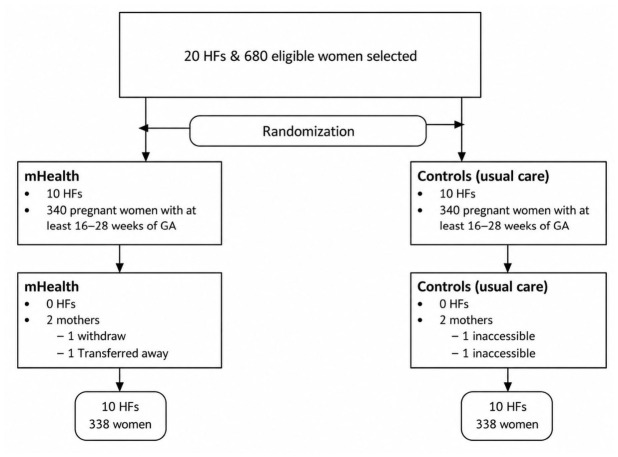
Diagrammatic sketch showing study procedures.

Clusters and Participants: After accounting for exclusions due to loss at follow-up, we analyzed 20 clusters and 676 participants (see Figure 2).

**Figure 3:**
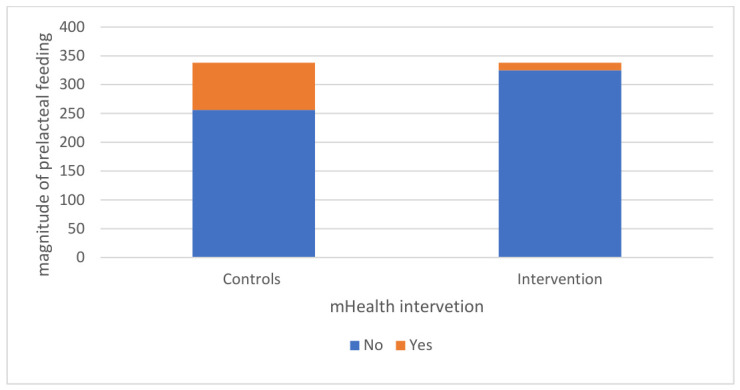
The distribution of prelacteal feeding among mothers using mHealth and standard care.

Figure 3 displays the distribution of prelacteal feeding among the mHealth and standard-care mothers. Prelacteal feeding was 26% among controls and 4% among mHealth mothers (Figure 3).

### Cluster Report

This trial includes 20 clusters (10 intervention and 10 control), with primary health facilities serving as the unit of randomization across separate districts. In the Ethiopian health system, primary health facilities are categorized by catchment population: primary hospitals (100,000 people), health centers (25,000 people), and health posts (5000 people). Each arm contains one primary hospital. Because the catchment population of each facility is known and the same number of women (35 per cluster) was randomized, the clusters were balanced in size. The estimated number of pregnancies in the country is 4%. Of the 340 women randomized per arm, 338 were available for analysis, indicating balanced follow-up across both arms (Figure 1). Only two women in each group did not commence the intervention and were recorded as lost at follow-up. As clustering strongly influenced the study outcome, multi-level analysis was used to account for this clustering effect.

Baseline Characteristics: There were no significant differences in age, residence, maternal education, gestational age, or maternal occupation between the control and intervention groups. The participants’ mean age was 28.15 ± 0.22, with a family size of 5.4 ± 0.08, number of children of 3.3 ± 0.07, and a gestational age of 6 ± 0.03 months at recruitment. More than two-thirds of the participants were from rural communities (68.04%). There is a significant difference in the number of children, family size, and religion between the control and intervention groups, with the control group having more children. Additionally, significant differences were observed in family size and religion (Table 1).

Primary Outcome: Table 2 presents different levels of the model, and Table 3 shows the model evaluation criteria.

The random effect analysis result

The null model showed a 32% variation in prelacteal feeding due to intra-cluster correlation variation. The ICC was reduced to 16% through the implementation of subsequent models. The remaining variation can be reduced by including additional cluster-level variables. The final (mixed-effect) model significantly improved performance from 1.54 to 0.63 (Table 2).

The fixed effect analysis result

The fixed-effect model identified a significant association of prelacteal feeding with maternal education, time to 1st PNC, ANC visit, partner education, and partner occupation (Table 3).

The mixed-effect analysis result

The study found that prelacteal feeding was significantly more prevalent in controls than in intervention groups (AOR = 0.19, 95%CI: 0.06–0.58, *p* < 0.01). The study found that 69% of mothers with college-level education reduced prelacteal feeding (AOR = 0.31, 95%CI: 0.09–0.99). Mothers with higher ANC visits have a 72% lower prelacteal feeding practice (AOR = 0.28, 95%CI: 0.21–0.39, *p* < 0.001). Mothers with partners’ educational level of college and above have 89% reduced prelacteal feeding (AOR= 0.11, 95%CI: 0.02–0.71, *p* < 0.05). Mothers with delayed time to first PNC have slightly higher odds of prelacteal feeding (Table 3).

Secondary Outcomes

Frequency of Antenatal Care (Anc) for the Current Pregnancy

Random effects model

The initial multi-level Poisson regression model has a log-likelihood (LL) of −1246, Akaike Information Criterion (AIC) of 2497, and Bayesian Information Criterion (BIC) of 2506. The final model demonstrated improved fit, with a log-likelihood (LL) of −1240.56, deviance of 2481.00, AIC of 2493.12, and BIC of 2520.22 (Table 4).

Fixed effects (coefficients)

Compared to the control group, the intervention group had a higher incidence of ANC visits (IRR = 1.15, 95% CI: 1.06–1.25). The maternal occupations (housewife) significantly reduce the incidence of ANC visits (IRR = 0.9, 95% CI: 0.83–0.99). Additionally, mothers with higher ANC visits had an increased incidence of initiating breastfeeding in the first hour after birth (IRR = 1.13, 95% CI: 1.03–1.25) (Table 4 footnote).

NB: Slight overdispersion was observed in the result; comparing the negative binomial model with the Poisson model delivered the same results. Thus, we conclude no or less meaningful dispersion.

Time to Postnatal Care Visit (Pnc) for the Current Child

The postnatal care visits were delayed by 2.05 days for women in the control group. Compared to women in families with one child, PNC days were reduced for families with 2–3 children (β = −1.08) and 4–6 children (β = −1.35). Compared to the Health Extension Worker (HEW) service provider, PNC visit days were reduced for nurses (β = −1.35), midwives (β = −1.10), and health officers (β = −1.10) (Table 5).

NB: The time for the first postnatal care visit was initially measured in hours and later converted to days. A shorter time to the first postnatal care visit reflects earlier and better PNC-seeking behavior.

## 4. Discussion

The mHealth intervention significantly reduced the likelihood of prelacteal feeding, with mothers in the intervention group 81% less likely to engage in the practice than those in the control group. This result aligns with previous studies demonstrating the effectiveness of mHealth tools in promoting optimal breastfeeding practices by addressing misconceptions and providing real-time reminders and health education [36,37]. The findings suggest that mHealth can bridge communication gaps in resource-limited settings, reinforcing messages delivered during antenatal care (ANC) and postnatal care (PNC) visits. The baseline characteristics were balanced between the groups; however, some variables, such as religion, number of children, and family size, slightly showed differences. The model evaluations presented in Table 2 indicate the highest group-level variance (1.54) and intra-cluster correlation coefficient (ICC = 0.32), indicating significant variability before adding predictors. The log-likelihood ratio, deviance, AIC, and BIC change in Table 2 indicate a parsimonious model.

Higher maternal education levels were strongly associated with reduced prelacteal feeding practices, with mothers having secondary education or above significantly less likely to engage in prelacteal feeding. This finding is consistent with the literature, highlighting maternal education as a critical determinant of breastfeeding practices [38,39]. Educated mothers may understand the risks associated with prelacteal feeding and the benefits of exclusive breastfeeding (EBF). Partner education at the college level and above reduces the likelihood of prelacteal feeding. The role of partner education in influencing maternal health decisions is well-documented, as partners often provide critical support and resources for breastfeeding practices [40]. Mothers who attended more antenatal care (ANC) visits experienced a 73% reduction in prelacteal feeding. In addition to mHealth, this reinforces the importance of consistent contact with healthcare providers, who can deliver tailored messages about breastfeeding and address misconceptions about prelacteal feeding [41]. Conversely, delays in the first PNC visit increased the odds of prelacteal feeding, suggesting that timely postnatal follow-up is critical for reinforcing breastfeeding practices [39,41]. In this regard, contact with a health provider in the immediate postpartum period could be vital.

For the secondary outcomes, our study found that women in the control group had significantly lower incidence of ANC visits than those in the intervention group. The findings align with the previous African and global literature [12,42,43,44,45]. This might show that mHealth motivates mothers to take more ANC visits. Maternal occupation (housewives) reduces the incidence of ANC visits. Studies consistently acknowledge the impact of maternal occupation on ANC, emphasizing the importance of early interventions to improve care-seeking behaviors among different occupational statuses [46,47]. Housewives may be uneducated and also take responsibility for their family in addition to pregnancy, which can reduce their ANC visits. Initiation of breastfeeding within the first hour after birth demonstrated a significant non-casual association. Breastfeeding initiation occurs after ANC attendance in the causal pathway; the observed association between the two variables reflects shared determinants of health-seeking behavior. Women who attended ANC were more likely to practice early breastfeeding, rather than early breastfeeding influencing ANC attendance. This association should be interpreted as a correlation between two positive maternal health practices, not as a causal effect. It is only a non-causal association rather than a predictor of ANC frequency.

The control group mothers experience a delay in taking the first PNC. A prior study found that mHealth enhanced postnatal care by positively influencing mothers’ behavior during ANC [48] This indicates that mothers’ behavior can be influenced during ANC for better PNC. Nurses, midwives, and health officers influenced (reduced) the time to first PNC compared to HEW professionals. HEWs are the backbone in providing basic maternal and child health care in Ethiopian rural communities; however, they are less educated and cannot be directly compared to other professions in specific activities, including information provision [49]. This may indicate that community health services in Ethiopia might need other professional support. Comparing women in families with one child and seven or more children, families with 2–3 and 4–6 children had reduced PNC time. Large family size is among the documented factors that limit maternal and child health services, including postnatal care [50]. Mothers with less experience at their first birth may become experienced, but having a large number of children equally reduces service uptake.

## 5. Conclusions

This study examined the impact of mHealth in a cluster-randomized controlled trial, focusing on key maternal and child health outcomes, including prelacteal feeding practices, time to postnatal care (PNC), and time to antenatal care (ANC). The findings, analyzed using statistical methods such as mixed-effects models, offer valuable insights into the impact of mHealth on the outcomes.

We observed that prelacteal feeding, time to PNC, and ANC frequency improved—indicating that mHealth may have contributed to timely healthcare-seeking behaviors among mothers. Prelacteal feeding, a risk factor for breastfeeding complications, is more common among less educated women, but less common among women with higher ANCs, suggesting potential intervention directions. Some women may also think breast milk is not sufficient for the baby and add complementary diets. However, mHealth provided information that enabled mothers to reduce prelacteal feeding.

Overall, this trial highlights the importance of contextually tailored interventions for improving maternal and neonatal health outcomes. The findings support the need for sustained efforts to promote timely access to healthcare and culturally sensitive practices in maternal and child health programs.

### 5.1. Implication of the Findings

These findings have several implications for public health interventions aimed at improving maternal and child health:

- Antenatal Care Promotion: The significant reduction in the odds of prelacteal feeding among mothers who attended ANC visits highlights the need for continued efforts to encourage frequent and early ANC attendance. Public health programs should focus on making ANC services more accessible, especially in rural areas, and reinforcing the importance of these visits for promoting optimal infant feeding practices.
- Targeted Interventions for Occupation: The stark differences in feeding practices based on maternal occupation suggest that targeted interventions may be necessary. For example, providing tailored health education and support for specific maternal occupations may help reduce harmful feeding practices. Engaging housewives in educational programs could further reduce prelacteal feeding practices.
- Contexts: mHealth is more effective in regions with limited-service provision and access challenges, while it shows minimal impact where service uptake is high. Before implementation, it is crucial to understand the context, socioeconomics, and sociodemographic characteristics of the target population.

### 5.2. Strengths and Limitations

This study has several strengths, including its robust cluster-randomized design and multi-level modeling to account for individual- and cluster-level variability. The study design allowed for a comprehensive analysis of the effects on key maternal and child health outcomes. Additionally, the reduction in intra-cluster correlation (ICC) from 32% to 16% in the mixed effects models demonstrates that including cluster-level variables significantly improved model fit and reduced unexplained variance.

However, some limitations must be acknowledged. Despite the use of multi-level models, residual confounding due to unmeasured factors such as socioeconomic status or detailed cultural practices can still exist. Additionally, the study’s findings may be context-specific, limiting their generalizability to other settings with different cultural or healthcare environments. The remaining cluster-level variance can be reduced by including more cluster-level variables.

### 5.3. Future Research

Future studies should explore the long-term effects of reduced prelacteal feeding on infant health outcomes and assess whether similar interventions can be effective in other settings with different cultural and healthcare dynamics. Additionally, interventions targeting less educated women and partners should be developed and evaluated to reduce the prevalence of harmful feeding practices.

## Figures and Tables

**Table 1:** Baseline characteristics of the study populations.

Variables		Intervention	Controls	χ^2^
		Number (%)	Number (%)	Df (*p*-Value)
GA at recruitment	16 wks	3 (0.89)	11 (3.25)	5.37 (0.147)
	20 wks	94 (27.81)	101 (29.88)	
	24 wks	224 (66.27)	208 (61.54)	
	28 wks	17 (5.03)	18 (5.33)	
Family size	2–3	84 (24.85)	75 (22.19)	22.28 (0.000)
	4–5	129 (38.17)	104 (30.77)	
	6–7	78 (23.08)	132 (39.05)	
	8+	47 (13.90)	27 (7.99)	
Maternal education	Read/write	37 (10.95)	26 (7.69)	3.76 (0.28)
	Primary school (1–8) grade	154 (45.56)	152 (44.97)	
	Secondary school (9–12) grade	93 (27.51)	111 (32.84)	
	College & above	54 (15.98)	49 (14.50)	
Residences	Rural	229 (67.75)	229 (67.75)	0.02 (0.86)
	Urban	109 (32.25)	109 (32.25)	
Maternal occupation	Farmer	136 (40.24)	154 (45.56)	2.07 (0.73)
	Housewife	112 (33.14)	104 (30.77)	
	Government employee	56 (16.57)	50 (14.79)	
	Marchant	26 (7.69)	24 (7.10)	
	Self-employed	8 (2.37)	6 (1.78)	
Number of children	1	51 (16.84)	49 (15.56)	19.93 (0.000)
	2–3	132 (43.56)	107 (33.97)	
	4–6	92 (30.36)	146 (46.35)	
	7+	28 (9.24)	13 (4.13)	
Religion	Ethiopian Orthodox Church	90 (26.63)	150 (44.38)	24.10 (0.000)
	Protestant	242 (71.60)	186 (55.03)	
	Muslims	6 (1.78)	2 (0.59)	

Key: Df = degree of freedom, and GA = gestational age.

**Table 2:** The evaluation of the multi-level model based on the various criteria.

Random Effect Model Comparison	Model 0	Model 1	Model 2	Model 3
Group-level Variance	1.54	0.38	0.85	0.63
Inter-cluster correlation (ICC)	0.32	0.10	0.20	0.16
Log-likelihood ratio (LLR)	−244.70	−234.23	−181.20	−180.68
Deviance	489.04	468.46	362.4	361.36
Proportional change in variance (PCV)	Ref	0.75	0.45	0.59
Media odds ratio (MOR)	3.30			
AIC	493.41	480.46	402.40	395.38
BIC	502.44	507.55	492.70	472.15

**Table 3:** The multi-level interaction of mobile phone text message intervention with study variables.

Variables		Model 0	Model 1	Model 2	Model 3
Maternal education	Read/write	-	-	1	1
	Primary school (1–8 grade)	-	-	0.48 (0.18–1.31)	
	Secondary school (9–12 grade)	-	-	0.29 (0.10–0.84) *	
	College & above	-	-	0.26 (0.08–0.85) *	0.31 (0.09–0.99) *
Partner occupation	Farmer	-	-	1	1
	Government employ	-	-	7.26 (1.50–34.95) *	
	Merchant	-	-	1.5 (0.59–3.91)	
	Self-employ	-	-	0.79 (0.17–3.5)	
	Others		-	0.40 (0.04–3.80)	
Partner Education	Read/write	-	-	1	
	Primary school(1–8 grade)	-	-	0.5 (0.11–2.15)	
	Secondary school (9–12 grade)	-	-	0.75 (0.18–3.10)	
	College & above	-	-	0.08 (0.01–0.56) *	0.11 (0.02–0.71) *
Ethnicity	Gamo/Gofa	-	-	1	
	Kore	-	-	1.96 (0.79–4.89)	
	Wolayta	-	-	1.31 (0.36–4.80)	
	Amhara	-	-	2.27 (0.57–8.89)	
Food before breastfeeding	No	-	-	1	
	Yes	-	-	1.64 (0.54–5.02)	
	I don’t know	-	-	0.52 (0.20–1.40)	
Time to 1st PNC		-	-	1.02 (1.00–1.05) *	1.00 (1.00–1.01) *
ANC		-	-	0.27 (0.19–0.38) ***	0.28 (0.21–0.39) ***
Bottle feeding	No	-	-	1	
	Yes	-	-	0.44 (0.16–1.15)	
Community and group-level variables					
Residences	Rural	-	1	-	
	Urban	-	1.13 (0.50–2.53)	-	
Treatment	Control	-	1	-	
	Intervention	-	0.09 (0.03–0.24) ***	-	0.19 (0.06–0.58) **
community education	Low	-	1	-	
	High	-	1.96 (0.83–4.61)	-	
Community occupation	No	-	1	-	
	Yes	-	1.60 (0.65–3.96)	-	

Key: * *p* < 0.05, ** *p* < 0.01, *** *p* < 0.001, and ANC = antenatal care.

**Table 4:** Frequency of ANC as a secondary outcome.

Variables	Categories	IRR (95% CI)
Treatment group	Controls	1
	Intervention	1.15 (1.06–1.25) ***
Maternal occupation	Farmer	1
	Housewife	0.90 (0.83–0.99) *
	Government employee	0.93 (0.83–1.05)
	Merchant	0.87 (0.73–1.02)
	Self-employed	0.99 (0.75–1.03)
BF initiation in 1 h	No	1
	Yes	1.13 (1.03–1.25) *

Key: * *p* < 0.05, and *** *p* < 0.001. Model fitness—null to final model: LL = −1240.56, deviance = 2481, AIC = 2493.12, and BIC = 2520.22.

**Table 5:** Time to postnatal care as a secondary outcome.

Variables	Categories	St Err	β-Coefficient (95% CI)
Treatment group	Controls	1	1
	Intervention	0.32	−2.05 (−2.66–−1.42) ***
Use media	No	1	1
	Yes	0.28	0.54 (−0.01–1.10)
Number of children	1		1
	2–3	0.40	−1.08 (−1.88–−0.29) *
	4–6	0.41	−1.35 (−2.15–−0.54) **
	7+	0.63	−0.46 (−1.72–0.78)
Provider	Nurse	0.45	−1.35 (−2.25–−0.45) **
	Midwife	0.38	−1.10 (−1.77–−0.27) **
	HO	0.43	−1.10 (−1.94–−0.24) *
	Doctors	0.60	−0.98 (−2.15–−0.19)
	HEWs	1	1

Key: * *p* < 0.05, ** *p* < 0.01, and *** *p* < 0.001. HO: health officers; HEWs: Health Extension Workers. The variation in outcome across groups is small (var (cons = 0.07), std. Err = 0.15), the variation residual is (var (Residual) = 11.45, Std. Err = 0.66), and log-likelihood = −1632, ICC = 0.01.

## Data Availability

The original contributions presented in this study are included in the article. Further inquiries can be directed to the corresponding author.

## Abbreviations

EBF: exclusive breastfeeding; BF: breastfeeding, HFs: health facilities; UNICEF: United Nations Children Emergency Fund; ANC: antenatal care; PNC: postnatal care; ICC: intra-cluster correlation; HEWs: Health Extension Workers; AIC: Akaike Information Criterion; BIC: Bayesian Information Criterion; GA: gestational age; Wks: weeks; ODK: Open Data Kit; AOR: adjusted odds ratio; AMU-IRB: Arba Minch University Institutional Review Board.
